# The effect of body mass index at diagnosis on clinical outcome in children with newly diagnosed acute lymphoblastic leukemia

**DOI:** 10.1038/bcj.2017.11

**Published:** 2017-02-17

**Authors:** H M Eissa, Y Zhou, J C Panetta, E K Browne, S Jeha, C Cheng, M V Relling, D Campana, C-H Pui, H Inaba

**Affiliations:** 1 Department of Oncology, St Jude Children’s Research Hospital, Memphis, TN, USA; 2 Department of Biostatistics, St Jude Children’s Research Hospital, Memphis, TN, USA; 3 Department of Pharmaceutical Sciences, St Jude Children’s Research Hospital, Memphis, TN, USA; 4 Department of Pediatric Medicine, St Jude Children’s Research Hospital, Memphis, TN, USA; 5 Department of Pediatrics, University of Tennessee Health Science Center, Memphis, TN, USA; 6 Department of Clinical Pharmacy, University of Tennessee Health Science Center, Memphis, TN, USA; 7 Department of Pediatrics, National University of Singapore, Singapore, Singapore

## Abstract

The impact of body mass index (BMI) at diagnosis on treatment outcome in children with acute lymphoblastic leukemia (ALL) is controversial. We studied 373 children with ALL enrolled on the Total XV study, which prospectively used minimal residual disease (MRD) for risk assignment. MRD on day 19 and at the end of remission induction (day 46), cumulative incidence of relapse/refractory disease (CIR), event-free survival (EFS) and overall survival (OS) were evaluated using sets of four, three and two subgroups based on BMI at diagnosis, along with BMI percentile change during remission induction. Higher BMI was associated with older age and higher treatment risk. There was no association between MRD on days 19 or 46 and BMI for four, three or two BMI subgroups (*P*>0.1 in all cases), nor was BMI associated with CIR or EFS. Obese patients had worse OS compared with non-obese (*P*=0.031) due to treatment-related mortality and less salvage after refractory disease or bone marrow relapse. No association between BMI change during remission induction and MRD, CIR, EFS or OS was seen. BMI at diagnosis does not predict poorer response or relapse in a contemporary MRD-directed ALL regimen. Improvements in supportive care and innovative, less-toxic frontline/salvage therapies are needed, especially for obese patients.

## Introduction

The 5-year survival rate for acute lymphoblastic leukemia (ALL), the most common childhood cancer, has risen over the past 4 decades to exceed 90%.^1, 2, 3^ This is the result of improved risk-directed protocol treatment and supportive care.^2^

In children with acute myeloid leukemia, obesity at diagnosis has been associated with worse outcomes.^4, 5^ However, in childhood ALL, the association between body weight at diagnosis and outcome is controversial.^6, 7, 8, 9^ In a Children’s Oncology Group study, children who were aged 10 years or older at diagnosis and were classified as obese because of a body mass index (BMI) in the 95th percentile or higher had a significantly higher relapse rate and worse event-free survival (EFS) when compared with non-obese children.^7^ Another study reported worse EFS for children who were obese (BMI ⩾95th percentile) or underweight (BMI <5th percentile) at diagnosis or for at least 50% of the time between the end of induction and the start of maintenance therapy, although BMI normalization during this period mitigated the risk.^8^ Obese or underweight status was also associated with increased risk of toxicity.^8^ Furthermore, a single-institution study of patients with B-ALL showed an association between obese or overweight (BMI ⩾85th percentile) status with positive end-of-induction minimal residual disease (MRD) and worse EFS.^9^ In contrast to the relation between BMI and treatment response observed in these three studies, an early study at St Jude Children’s Research Hospital (St Jude) showed that BMI at diagnosis did not affect outcome or treatment-related toxicity.^6^

Nearly 17% of children and adolescents in the United States are currently considered obese.^10, 11^ Therefore, we reassessed the impact of BMI on treatment response and survival in our recent patient cohort, consisting of children and adolescents with ALL treated on the St Jude Total XV protocol, which prospectively used MRD for risk assignment.^12, 13^

## Subjects and Methods

### Patients

Patients with newly diagnosed ALL treated on the Total XV protocol at St Jude between 2000 and 2007 were included in this study. Written informed consent was obtained from legal guardians and assent from patients as appropriate. This study was approved by the St Jude Institutional Review Board.

### BMI classification

BMI was calculated from height and weight. The SAS program for the CDC growth charts (http://www.cdc.gov/nccdphp/dnpao/growthcharts/resources/sas.htm) was used to calculate BMI percentiles for age and sex. Patients aged 2–18 years were defined as underweight, normal weight, overweight or obese according to whether their BMI was below the 5th percentile, in or above the 5th percentile but below the 85th percentile, in or above the 85th percentile but below the 95th percentile or in or above the 95th percentile, respectively.^14^ Patients younger than 2 years and those with Down syndrome were excluded from the analysis.

### Treatment

The Total XV protocol has been described elsewhere.^12^ Risk classification was based on the presenting characteristics (age, white blood cell count, immunophenotype and cytogenetics) and treatment response as measured by MRD level during remission induction (on day 19) and at the end of remission induction therapy (on or around day 46). After upfront methotrexate treatment, conventional remission induction therapy was initiated on day 4, consisting of a four-drug regimen (prednisone, vincristine, daunorubicin and L-asparaginase) followed by administration of cyclophosphamide, mercaptopurine and cytarabine. Patients with an MRD level of 1% or higher on day 19 received three additional L-asparaginase doses. Thereafter, patients received consolidation therapy (8 weeks) and continuation therapy (120 weeks for girls and 146 weeks for boys) based on their risk assignment.^12, 15^ Body surface area derived from actual body weight was used for the dosing of all systemic chemotherapeutic agents except vincristine, for which the maximum dose was capped at 2 mg.

Grade 3 and 4 treatment-related toxicities during Total XV protocol therapy were prospectively collected according to the National Cancer Institute Common Terminology Criteria for Adverse Events (CTCAE) version 3.0.

### Statistical methods

BMI at diagnosis was analyzed in four (underweight, normal, overweight and obese), three (underweight, normal and overweight/obese) and two (obese and non-obese) subgroups. BMI percentile changes from diagnosis to the end of induction were analyzed as a continuous variable.

The distribution of presenting features and MRD on day 19 (<1 vs ⩾1%) and at the end of induction (<0.01 vs ⩾0.01%) were compared across the groups by *χ*^2^-tests. Complete remission was defined as having <5% leukemic blasts with restoration of normal hematopoiesis. The cumulative incidence of refractory or relapsed disease (CIR) was estimated as described by Kalbfleisch and Prentice^16^ and compared by Gray’s method;^17^ second cancer or death (from any cause) were regarded as competing events. The EFS duration was measured from the date of complete remission to the date of first treatment failure (relapse, death or second malignant neoplasm) or the date of most recent follow-up. Failure to induce remission was considered an event at time zero. The overall survival (OS) duration was calculated from the date of diagnosis to the date of death or most recent follow-up. The EFS and OS distributions were estimated by the method of Kaplan and Meier^18^ and compared by the log-rank test.^19^ A multivariable Cox proportional hazards regression model was used to further assess the effects of obesity on EFS and OS.^20^

## Results

### Patients

**Figure 1:**
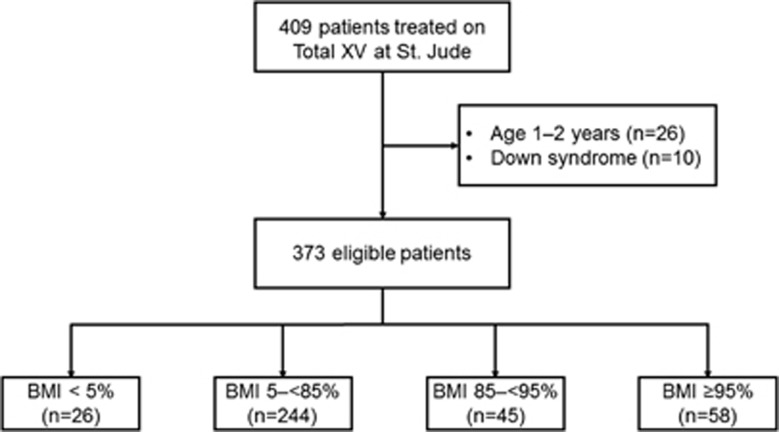
CONSORT diagram.

We reviewed the records of the 409 patients who were treated on the Total XV protocol at St Jude (Figure 1). Twenty-six patients aged between 1 and 2 years and 10 patients with Down syndrome were excluded, leaving 373 patients evaluable for this study. Of these 373 patients, 26 (7.0%) were underweight, 244 (65.4%) had a normal BMI, 45 (12.1%) were overweight and 58 (15.5%) were obese.

Among the four subgroups, higher BMI was associated with older age at diagnosis (*P*=0.008) and higher Total XV risk (*P*=0.041; Table 1). Similarly, when obese patients (*n*=58) were compared with non-obese patients (*n*=315) as two subgroups, the obese group was found to be significantly older (*P*=0.002) and had higher Total XV risk (*P*=0.009; Supplementary Table S1). For three subgroups (overweight and obese (*n*=103), normal (*n*=244) and underweight (*n*=26)), higher BMI was associated with older age (*P*=0.005), higher white blood cell count (*P*=0.037), T-cell phenotype (*P*=0.042) and higher Total XV risk (*P*=0.026; Supplementary Table S1).

### Minimal residual disease by BMI category

The association of BMI and MRD on day 19 of induction (<1 vs ⩾1%) and on day 46 (the end of induction; <0.01 vs ⩾0.01%) is shown in Supplementary Table S2. An MRD level of 1% or higher on day 19 and 0.01% or more on day 46 was significantly associated with high initial white blood cell counts (*P*=0.016 and 0.015, respectively), T-cell phenotype (*P*=0.004 and 0.007, respectively) and higher Total XV risk (*P*<0.001 for both). In addition, male sex (*P*=0.005) was associated with a day 19 MRD level of 1% or higher, and age 10 years or older at diagnosis was associated with a day 46 MRD level of 0.01% or higher (*P*=0.017). However, there was no difference in MRD levels between BMI categories when compared as four subgroups (*P*=0.428 and 0.177 on days 19 and 46, respectively), three subgroups (*P*=0.278 and 0.113) or two subgroups (*P*=0.758 and 0.173; Table 1; Supplementary Table S2).

### Outcome by BMI category

**Figure 2:**
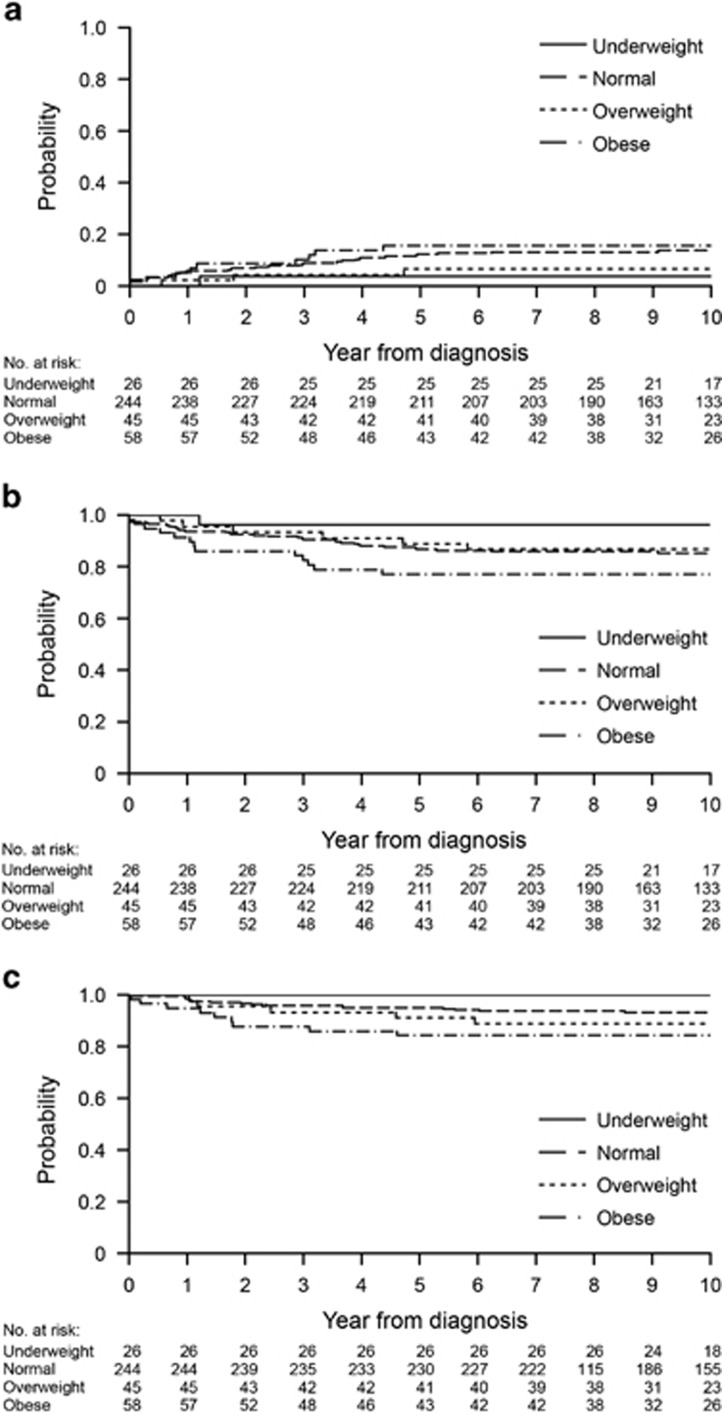
Clinical outcome according to BMI category. Kaplan–Meier curves are shown for (**a**) the CIR, (**b**) EFS and (**c**) OS. BMI categories are underweight (<5th percentile), normal (5th to <85th percentile), overweight (85th to <95th percentile) and obese (⩾95th percentile).

The median follow-up time among survivors was 10.1 years (range, 3.2–14.1 years). Table 2 shows the 10-year CIR, EFS and OS based on clinical characteristics. Worse CIR was associated with male sex (*P*=0.006), higher Total XV risk (*P*<0.001) a day 19 MRD of 1% or higher (*P*<0.001) and a day 46 MRD level of 0.01% or higher (*P*<0.001). There was no association between CIR and the four BMI subgroups (underweight, 3.8±3.8% normal weight, 13.9±2.3% overweight, 6.7±3.8% and obese, 15.7±4.9%, *P*=0.257; Figure 2a), three subgroups (*P*=0.349) or two subgroups (*P*=0.400).

Worse EFS was associated with male sex (*P*=0.029), T-cell phenotype (*P*=0.006), higher Total XV risk (*P* <0.001), a day 19 MRD level of 1% or higher (*P*<0.001) and a day 46 MRD level of 0.01% or higher (*P* <0.001; Table 2). There was no significant association between EFS and BMI when analyzed as four subgroups (underweight, 96.2±4.7% normal weight, 85.3±3.3% overweight, 86.7±6.9% and obese, 77.1±7.9%, *P*=0.159; Figure 2b) or three subgroups (*P*=0.208); obese patients tended to have worse EFS (77.1±7.9%) when compared with non-obese patients (86.4±2.8%) as two subgroups (*P*=0.054). In multivariable Cox proportional hazards regression models adjusted for Total XV risk, sex, race and receipt of a hematopoietic stem cell transplant (HSCT) as a time-dependent variable, there were no significant differences between the four BMI subgroups (*P*=0.378), three subgroups (*P*=0.427) or two subgroups (*P*=0.148; Table 3). Higher Total XV risk and HSCT were associated with worse EFS for all BMI subgroups in this model (*P* ⩽0.003 for all cases).

Worse OS was associated with age of 10 years or older at diagnosis (*P*<0.001), T-cell phenotype (*P*=0.012), higher Total XV risk (*P*<0.001) and MRD on days 19 and 46 (*P*<0.001 for both; Table 2). Obese patients did not have worse OS in the analysis based on four subgroups (underweight, 100±0.0% normal weight, 93.3±2.2% overweight, 88.9±6.3% and obese, 84.3±6.7% *P*=0.152; Figure 2c) or three subgroups (*P*=0.105) but did have worse OS in the analysis based on two subgroups (obese, 84.3±6.7% vs non-obese, 93.2±2.0% *P*=0.019). After adjusting for Total XV risk, race and HSCT as a time-dependent variable, there was no difference in OS among four BMI subgroups (*P*=0.192) or three subgroups (*P*=0.131); however, obese patients still had worse survival in the analysis of two subgroups (*P*=0.031; Table 3). In this model, higher Total XV risk and HSCT were again associated with worse OS among all subgroups (all *P*⩽0.010).

### Effect of BMI percentile change

The effect of BMI percentile change between diagnosis and the end of induction on MRD, CIR, EFS and OS was evaluated as a continuous variable. MRD levels on days 19 and 46 were not associated with BMI percentile change (*P*=0.226 and 0.989, respectively) and neither were CIR, EFS or OS (*P*=0.607, 0.190 and 0.117, respectively; Supplementary Table S3).

### Events, death and toxicities in obese and non-obese patients

As obese patients had worse OS when compared with non-obese patients (when analyzed as two subgroups), we evaluated the details of the 55 events and 30 deaths. Thirteen events, including 9 deaths, occurred in 58 obese patients, and 42 events, including 21 deaths, occurred in 315 non-obese patients (Table 4; Supplementary Table S4).

Eight of the 13 events in 58 obese patients were due to leukemia relapses. Although all 3 patients with isolated central nervous system relapse were salvaged, only 1 of 5 patients with bone marrow or combined relapse remains alive. Four of the 58 patients (6.9%) died from therapy-related toxicity: 1 each from *Bacillus cereus* sepsis, systemic infection with *Clostridium* species and *Bacteroides caccae*, liver failure and transplant-associated complications. One patient died in a motor vehicle accident after completing therapy.

Among the 42 events in 315 non-obese patients, 36 were due to refractory disease or relapse; all isolated CNS (*n*=6) and testicular (*n*=2) relapses were salvaged, as were 13 of 28 patients with refractory disease or bone marrow/combined relapses. Two patients died of second malignant neoplasms (acute myeloid leukemia and high-grade glioma). Four of 315 patients (1.3%) died from therapy-related toxicity, namely *B. cereus* sepsis (*n*=1) or transplant-associated complications (*n*=3).

The overall occurrence of grade 3 and 4 toxicities during the induction and post-induction phases in obese and non-obese patients was examined (Table 4). There was no difference between the two subgroups in terms of the number of patients who developed toxicities during induction (*P*=0.732) or post induction (*P*=0.276).

## Discussion

In this study, we evaluated the association between BMI at diagnosis, early treatment response as assessed by MRD and long-term outcome in children with ALL. Higher BMI was associated with older age and adverse presenting features. There were no associations between BMI at diagnosis and MRD on days 19 or 46 of induction. Furthermore, we observed no significant association between BMI and CIR or EFS, although obese patients had worse OS when compared with non-obese patients.

Persistent MRD in the bone marrow is an important early prognostic indicator^21, 22^ and was strongly associated with CIR, EFS and OS in this study. The lack of association between BMI and MRD in our study contrasts with the previously reported association between obese and overweight status and positive MRD at the end of induction and worse EFS.^9^ However, that study had relatively short follow-up (median, 1.9 years), was limited to B-ALL patients and included only 198 patients treated on multiple protocols without uniform treatment modification for positive MRD. Moreover, 79.3% of the patients were Hispanic, an ethnicity associated with a higher prevalence of obesity.^23^ In addition, Hispanic ethnicity is associated with a worse outcome, possibly attributable to the greater prevalence of high-risk disease, such as *BCR-ABL1*-like ALL, or poorer compliance to oral mercaptopurine.^24, 25, 26^ However, in our current protocol, Hispanic patients were a minority (*n*=22, 5.9%, data not shown), both B- and T-cell ALL patients were treated and there was a longer follow-up. Other than the randomized duration of methotrexate administration (4 vs 24 h) before starting induction, our patients received an identical regimen until day 19. Patients with MRD levels of 1% or higher on day 19 received three additional L-asparaginase doses. We also evaluated MRD responses by using continuous BMI percentiles but observed no significant differences (data not shown).

Consistent with the lack of association with early treatment response, we found no association between BMI and CIR or EFS. Butturini *et al.*^7^ evaluated morphologic bone marrow responses on day 7 and at the end of induction and found no significant difference between obese and non-obese patients, although the relapse rate was worse in obese patients. Morphologic evaluation is insufficiently sensitive to evaluate residual disease and provide response-based treatment modification.^22^ In the Total XV protocol, post-induction treatment was adjusted based on the presenting features and MRD levels, with an excellent 5-year CIR of 9.3% this might have mitigated the difference in CIR and EFS among the BMI subgroups.^12, 15^ The other two studies showing worse EFS in obese patients did not evaluate CIR.^8, 9^ Factors other than relapsed/refractory disease might have contributed to the worse EFS; in one study, EFS was worse in obese and overweight patients irrespective of the end-of-induction MRD.^9^

Our results are consistent with those of a previous study at our center, which found that BMI at diagnosis was not associated with worse CIR or EFS.^6^ Changes in BMI *Z*-score during induction, as well as BMI *Z*-score at diagnosis, are significant predictors of obesity at the end of therapy.^27^ We observed no association between changes in BMI *Z*-score and MRD, CIR, EFS or OS.

OS was worse in obese patients in multivariate analysis. Therefore, we evaluated patients who experienced events by focusing on treatment-related toxicities and salvage patterns after relapses. Treatment-related death was more common in obese patients (of whom 4 of 58 (6.9%) died) than in non-obese patients (of whom 4 of 315 (1.3%) died), although the incidences of CTCAE grade 3 or 4 toxicities were similar in these subgroups during the Total XV protocol therapy. The Total XV protocol did not use cranial irradiation for CNS control during frontline treatment, and all patients experiencing CNS relapse were salvaged with systemic chemotherapy with cranial irradiation, regardless of their BMI category. However, among patients with bone marrow and combined (bone marrow and extramedullary) relapses or refractory disease, only 1 of 5 obese patients (20%) was salvaged, compared with 13 of 28 non-obese patients (46.4%). Our small number of patients precludes definite conclusions; however, obese patients might have less tolerance for severe life-threatening toxicities and intensive salvage therapy for refractory or relapsed disease. Efforts during therapy to normalize the patient’s weight category, which mitigated worse prognosis in one study,^8^ as well as close monitoring for supportive care, should be considered. In cases of refractory/relapsed disease, efforts should be made to minimize intensive chemotherapy, including HSCT, by incorporating immunotherapy or molecular targeting agents.^1, 2^

Although our study was retrospective and included relatively few patients, all were treated on a contemporary regimen, prospectively using MRD as a basis for risk assignment. Other descriptors of body fat/lean mass content, including skin fold assessment and waist-to-height ratio,^28^ are increasingly recognized as being more accurate than BMI, but BMI remains the most widely used descriptor of obesity as it is readily available, inexpensive and easily transferable to clinical practice.

In conclusion, our results indicate that in the context of contemporary MRD-directed therapy, obesity at diagnosis is not an indicator for poor response or relapse in children or adolescents with ALL. However, further improvements in supportive care and innovative, less-toxic frontline and salvage therapies are needed, especially for obese patients.

## Figures and Tables

**Table 1:** Patient characteristics based on BMI

*Patient characteristics*	*Total (*n=*373)*	*BMI subgroups*				P*-value*
		*BMI <5% (*n=*26)*	*BMI 5–85% (*n=*244)*	*BMI 85–95% (*n=*45)*	*BMI* ⩾*95% (*n=*58)*	
*Race,* n *(%)*						0.754
White	265 (71.1)	21 (80.8)	173 (70.9)	29 (64.4)	42 (72.4)	
Black	68 (18.2)	3 (11.5)	43 (17.6)	12 (26.7)	10 (17.2)	
Other	40 (10.7)	2 (7.7)	28 (11.5)	4 (8.9)	6 (10.4)	
						
*Age at diagnosis (years),* n *(%)*						**0.008**
2 to <10	267 (71.6)	20 (76.9)	186 (76.2)	29 (64.4)	32 (55.2)	
⩾10	106 (28.4)	6 (23.1)	58 (23.8)	16 (35.6)	26 (44.8)	
						
*Sex,* n *(%)*						0.296
Male	214 (57.4)	15 (57.7)	138 (56.6)	22 (48.9)	39 (67.2)	
Female	159 (42.6)	11 (42.3)	106 (43.4)	23 (51.1)	19 (32.8)	
						
*WBC,* n *(%)*						0.097
<50 × 10^9^/l	272 (72.9)	22 (84.6)	186 (76.2)	29 (64.4)	35 (60.3)	
50–100 × 10^9^/l	54 (14.5)	3 (11.5)	32 (13.1)	7 (15.6)	12 (20.7)	
⩾100 × 10^9^/l	47 (12.6)	1 (3.9)	26 (10.7)	9 (20.0)	11 (19.0)	
						
*Immunophenotype,* n *(%)*						0.095
B cell	310 (83.1)	24 (92.3)	208 (85.2)	34 (75.6)	44 (75.9)	
T cell	63 (16.9)	2 (7.7)	36 (14.8)	11 (24.4)	14 (24.1)	
						
*HSCT*						0.373
Yes	30 (8.0)	0 (0.0)	23 (9.4)	3 (6.7)	4 (6.9)	
No	343 (92.0)	26 (100.0)	221 (90.6)	42 (93.3)	54 (93.1)	
						
*Total XV risk,* n *(%)*						**0.041**
Low	181 (48.5)	16 (61.5)	125 (51.2)	21 (46.7)	19 (32.8)	
Standard	163 (43.7)	9 (34.6)	96 (39.4)	22 (48.9)	36 (62.1)	
High	29 (7.8)	1 (3.9)	23 (9.4)	2 (4.4)	3 (5.2)	
						
*MRD on day 19,* n *(%)*						0.428
<1%	289 (77.5)	22 (84.6)	183 (75.0)	38 (84.4)	46 (79.3)	
⩾1%	76 (20.4)	3 (11.5)	55 (22.5)	7 (15.6)	11 (19.0)	
No data	8 (2.1)	1 (3.9)	6 (2.5)	0 (0.0)	1 (1.7)	
						
*MRD at end of induction,* n *(%)*						0.177
<0.01%	299 (80.2)	24 (92.3)	189 (77.5)	36 (80.0)	50 (86.2)	
⩾0.01%	69 (18.5)	2 (7.7)	52 (21.3)	8 (17.8)	7 (12.1)	
No data	5 (1.3)	0 (0)	3 (1.2)	1 (2.2)	1 (1.7)	

Abbreviations: BMI, body mass index; HSCT, hematopoietic stem cell transplant; MRD, minimal residual disease; WBC, white blood cells. *P*-values less than 0.05 are shown in bold.

**Table 2:** Cumulative incidence of refractory/relapsed disease, event-free survival and overall survival

*Characteristics*	N	*10-year CIR±s.e.*		*10-year EFS±s.e.*		*10-year OS±s.e.*	
		*%*	P*-value*	*%*	P*-value*	*%*	P*-value*
*Race*			0.128		0.055		0.461
White	265	10.3±1.9		87.8±2.8		92.8±2.2	
Black	68	19.1±4.8		76.5±8.3		87.7±5.8	
Other	40	16.2±6.3		81.3±8.5		92.5±5.3	
							
*Age at diagnosis (years)*			0.610		0.054		**<0.001**
2 to <10	267	12.2±2.0		87.0±2.9		95.0±1.8	
⩾10	106	13.5±3.4		79.7±5.8		83.7±5.1	
							
*Sex*			**0.006**		**0.029**		0.148
Male	214	16.8±2.6		81.3±3.8		89.9±2.8	
Female	159	7.0±2.0		89.8±3.5		94.3±2.6	
							
*WBC*			0.686		0.328		0.342
<50 × 10^9^/l	272	12.8±2.1		86.1±3.0		92.8±2.2	
50–100 × 10^9^/l	54	9.4±4.0		84.9±6.7		90.7±5.3	
⩾100 × 10^9^/l	47	14.9±5.3		78.4±8.1		87.0±6.5	
							
*Immunophenotype*			0.069		**0.006**		**0.012**
B cell	310	11.3±1.8		87.1±2.8		93.4±2.0	
T cell	63	19.0±5.0		74.3±7.1		83.9±6.0	
							
*Total XV risk*			**<0.001**		**<0.001**		**<0.001**
Low	182	5.3±1.8		94.1±2.6		97.6±1.6	
Standard	163	15.5±2.9		81.2±4.2		88.7±3.3	
High	29	41.4±9.4		48.3±13.1		72.4±10.5	
							
*MRD on day 19*			**<0.001**		**<0.001**		**<0.001**
<1%	289	11.3±1.8		91.6±2.3		96.0±1.6	
⩾1%	76	19.0±5.0		60.2±7.9		77.2±6.2	
							
*MRD at end of induction*			**<0.001**		**<0.001**		**<0.001**
<0.01%	299	9.0±1.7		89.6±2.6		95.5±1.7	
⩾0.01%	69	26.1±5.3		66.6±7.7		78.2±6.3	
							
*BMI (4 subgroups)*			0.257		0.159		0.152
<5%	26	3.8±3.8		96.2±4.7		100±0.0	
5 to <85%	244	13.9±2.3		85.3±3.3		93.3±2.2	
85 to <95%	45	6.7±3.8		86.7±6.9		88.9±6.3	
⩾95%	58	15.7±4.9		77.1±7.9		84.3±6.7	
							
*BMI (3 subgroups)*			0.349		0.208		0.105
<5%	26	3.8±3.8		96.2±4.7		100±0.0	
5 to <85%	244	13.9±2.3		85.3±3.3		93.3±2.2	
⩾85%	103	11.7±3.2		81.3±5.4		86.3±4.7	
							
*Obese vs non-obese*			0.400		0.054		**0.019**
⩾95%	58	15.7±4.9		77.1±7.9		84.3±6.7	
Other	315	12.0±1.9		86.4±2.8		93.2±2.0	

Abbreviations: BMI, body mass index; CIR, cumulative incidence of refractory disease/relapse; EFS, event-free survival; MRD, minimal residual disease; OS, overall survival; WBC, white blood cells. *P*-values less than 0.05 are shown in bold.

**Table 3:** Multivariate analysis for event-free survival and overall survival

*Characteristics*	*BMI 4 subgroups*	*BMI 3 subgroups*	*Obese vs non-obese*
*Event-free survival*			
Total XV risk	**0.001**	**0.001**	**0.001**
Standard/high vs low	3.48 (1.71–7.14)	3.53 (1.73–7.25)	3.52 (1.72–7.19)
Sex	0.127	0.106	0.121
Female vs male	0.63 (0.35–1.14)	0.61 (0.34–1.11)	0.63 (0.35–1.13)
Race	0.138	0.155	0.123
Black vs white	1.84 (1.00–3.39)	1.81 (0.984–3.32)	1.88 (1.02–3.45)
Others vs white	1.42 (0.62–3.24)	1.40 (0.61–3.20)	1.42 (0.62–3.24)
HSCT^a^	**0.002**	**0.003**	**0.001**
Yes vs no	3.30 (1.53–7.10)	3.24 (1.50–6.97)	3.48 (1.62–7.46)
BMI	0.378	0.427	0.148
Categories^b^	0.23 (0.03–1.77; 1 vs 4)	0.28 (0.04–2.12; 1 vs 3+4)	0.63 (0.33–1.18; 1+2+3 vs 4)
	0.67 (0.35–1.29; 2 vs 4)	0.83 (0.47–1.46; 2 vs 3+4)	
	0.58 (0.22–1.54; 3 vs 4)		
			
*Overall survival*			
Total XV risk	**0.010**	**0.009**	**0.009**
Standard/high vs low	4.20 (1.41–12.50)	4.33 (1.45–12.82)	4.35 (1.46–12.99)
Race	0.819	0.819	0.804
Black vs white	1.33 (0.58–3.10)	1.32 (0.57–3.05)	1.37 (0.59–3.19)
Others vs white	0.94 (0.27–3.22)	0.91 (0.27–3.14)	0.98(0.29–3.34)
HSCT^a^	**<0.001**	**0.001**	**<0.001**
Yes vs no	4.67 (1.98–11.02)	4.53 (1.93–10.65)	4.85 (2.06–1.41)
BMI	0.192	0.131	**0.031**
Categories^b^	0.00 (0.00–0.00; 1 vs 4)	0.00 (0.00–0.00; 1 vs 3+4)	0.41 (0.19–0.92; 1+2+3 vs 4)
	0.40 (0.17–0.93; 2 vs 4)	0.47 (0.23–0.98; 2 vs 3+4)	
	0.67 (0.22–2.03; 3 vs 4)		

Abbreviations: BMI, body mass index; HSCT, hematopoietic stem cell transplant. For each clinical characteristic, the *P*-value followed by the hazard ratio with 95% confidence interval is shown.

a HSCT was considered as a time-dependent variable.

b BMI categories: 1, underweight; 2, normal weight; 3, overweight; 4, obese. BMI 4 subgroups: underweight; normal weight; overweight; and obese. BMI 3 subgroups: underweight; normal weight; and overweight+obese. *P*-values less than 0.05 are shown in bold.

**Table 4:** Events, death and overall grade 3 and 4 toxicities during induction and post induction in obese and non-obese patients

	*Events and deaths*		
	*Total (*n=*373)*	*Non-obese (*n=*315)*	*Obese (*n=*58)*
*Events,* n *(%)*	55	42 (13.3)	13 (22.4)
Relapse/refractory disease	44	36 (11.4)	8 (13.8)
Bone marrow disease	33	28 (8.9)	5 (8.6)
Extramedullary disease	11	8 (2.5)	3 (5.2)
Treatment-related mortality	8	4 (1.3)	4 (6.9)
Other	3	2 (0.6)	1 (1.7)
Deaths, *n* (%)	30	21 (6.7)	9 (15.5)

	*Grade 3 and 4 toxicities*			
	*Total (*n=*373)*	*Non-obese (*n=*315)*	*Obese (*n=*58)*	P*-value*
*Induction*				
Patients, *n* (%)	250 (67.0)	210 (66.7)	40 (69.0)	0.732
Episodes, *n*	515	418	97	
				
*Post induction*				
Patients, *n* (%)	362 (97.1)	307 (97.5)	55 (94.8)	0.276
Episodes, *n*	2925	2416	509	
