# Supplementary material for: The effect of body mass index at diagnosis on clinical outcome in children with newly diagnosed acute lymphoblastic leukemia
Source: Blood Cancer J. 2017 Feb 17;7(2):e531–. doi: 10.1038/bcj.2017.11 (PMC5533940; doi:10.1038/bcj.2017.11)
Supplement: Supplementary Information [file bcj201711x1.docx]

**Supplementary Table S1.** Patient characteristics by 2 and 3 BMI subgroups

| **Characteristics** | | **Total**  **(n = 373)** | **BMI (2 subgroups)** | | | **BMI (3 subgroups)** | | | |
| --- | --- | --- | --- | --- | --- | --- | --- | --- | --- |
|  |  |  | **Non-obese**  **(n = 315)** | **Obese**  **(n = 58)** | ***P*-value** | **BMI < 5%**  **(n = 26)** | **BMI 5%–85%**  **(n = 244)** | **BMI ≥ 85%**  **(n = 103)** | ***P*-value** |
| **Race** | | |  |  | 0.922 |  |  |  | 0.630 |
| White | | 265 (71.1) | 223 (70.8) | 42 (72.4) |  | 21 (80.8) | 173 (70.9) | 71 (68.9) |  |
| Black | | 68 (18.2) | 58 (18.4) | 10 (17.2) |  | 3 (11.5) | 43 (17.6) | 22 (21.4) |  |
| Other | | 40 (10.7) | 34 (10.8) | 6 (10.4) |  | 2 (7.7) | 28 (11.5) | 10 (9.7) |  |
| **Age at diagnosis (years), n (%)** | | |  |  | **0.002** |  |  |  | **0.005** |
| 2 to < 10 | | 267 (71.6) | 235 (74.6) | 32 (55.2) |  | 20 (76.9) | 186 (76.2) | 61 (59.2) |  |
| ≥ 10 | | 106 (28.4) | 80 (25.4) | 26 (44.8) |  | 6 (23.1) | 58 (23.8) | 42 (40.8) |  |
| **Sex, n (%)** | | |  |  | 0.098 |  |  |  | 0.900 |
| Male | | 214 (57.4) | 175 (55.6) | 39 (67.2) |  | 15 (57.7) | 138 (56.6) | 61 (59.2) |  |
| Female | | 159 (42.6) | 140 (44.4) | 19 (32.8) |  | 11 (42.3) | 106 (43.4) | 42 (40.8) |  |
| **WBC, n (%)** | | |  |  | 0.063 |  |  |  | **0.037** |
| < 50 × 10^9^/L | | 272 (72.9) | 237 (75.3) | 35 (60.3) |  | 22 (84.6) | 186 (76.2) | 64 (62.1) |  |
| 50–100 × 10^9^/L | | 54 (14.5) | 42 (13.3) | 12 (20.7) |  | 3 (11.5) | 32 (13.1) | 19 (18.5) |  |
| ≥ 100 × 10^9^/L | | 47 (12.6) | 36 (11.4) | 11 (19.0) |  | 1 (3.9) | 26 (10.7) | 20 (19.4) |  |
| **Immunophenotype, n (%)** | | |  |  | 0.109 |  |  |  | **0.042** |
| B cell | | 310 (83.1) | 266 (84.4) | 44 (75.9) |  | 24 (92.3) | 208 (85.2) | 78 (75.7) |  |
| T cell | | 63 (16.9) | 49 (15.6) | 14 (24.1) |  | 2 (7.7) | 36 (14.8) | 25 (24.3) |  |
| **HSCT** | | |  |  | 0.727 |  |  |  | 0.210 |
| Yes | | 30 (8.0) | 26 (8.3) | 4 (6.9) |  | 0 (0.0) | 23 (9.4) | 7 (6.8) |  |
| No | | 343 ( 92.0) | 289 ( 91.7) | 54 ( 93.1) |  | 26 (100.0) | 221 (90.6) | 96 (93.2) |  |
| **Total XV risk, n (%)** | | |  |  | **0.009** |  |  |  | **0.026** |
| Low | 181 (48.5) | | 162 (51.4) | 19 (32.8) |  | 16 (61.5) | 125 (51.2) | 40 (38.8) |  |
| Standard | 163 (43.7) | | 127 (40.3) | 36 (62.1) |  | 9 (34.6) | 96 (39.4) | 58 (56.3) |  |
| High | 29 (7.8) | | 26 (8.3) | 3 (5.2) |  | 1 (3.9) | 23 (9.4) | 5 (4.9) |  |
| **MRD on day 19, n (%)** | | |  |  | 0.758 |  |  |  | 0.278 |
| < 1% | 289 (77.5) | | 243 (77.2) | 46 (79.3) |  | 22 (84.6) | 183 (75.0) | 84 (81.5) |  |
| ≥ 1% | 76 (20.4) | | 65 (20.6) | 11 (19.0) |  | 3 (11.5) | 55 (22.5) | 18 (17.5) |  |
| No data | 8 (2.1) | | 7 (2.2) | 1 (1.7) |  | 1 (3.8) | 6 (2.5) | 1 (1.0) |  |
| **MRD at end of induction, n (%)** | | |  |  | 0.173 |  |  |  | 0.113 |
| < 0.01% | 299 (80.2) | | 249 (79.0) | 50 (86.2) |  | 24 (92.3) | 189 (77.5) | 86 (83.5) |  |
| ≥ 0.01% | 69 (18.5) | | 62 (19.7) | 7 (12.1) |  | 2 (7.7) | 52 (21.3) | 15 (14.6) |  |
| No data | 5 (1.3) | | 4 (1.3) | 1 (1.7) |  | 0 (0.0) | 3 (1.2) | 2 (1.9) |  |

Abbreviations: BMI, body mass index; WBC, white blood cells; HSCT, hematopoietic stem cell transplant; MRD, minimal residual disease.

**Supplementary Table S2.** Patient characteristics and minimal residual disease on days 19 and 46

| **Characteristics** | **MRD on Day 19** | | | | **MRD at end of Induction** | | | |
| --- | --- | --- | --- | --- | --- | --- | --- | --- |
|  | **Total**  **(n = 365)** | **< 1%**  **(n = 289)** | **≥ 1%**  **(n = 76)** | ***P*-value** | **Total**  **(n = 368)** | **< 0.01%**  **(n = 299)** | **≥ 0.01%**  **(n = 69)** | ***P*-value** |
| **Race, n (%)** |  |  |  | 0.692 |  |  |  | 0.306 |
| White | 259 (70.9) | 208 (72.0) | 51 (67.1) |  | 262 (71.2) | 218 (72.9) | 44 (63.8) |  |
| Black | 66 (18.1) | 50 (17.3) | 16 (21.1) |  | 66 (17.9) | 51 (17.1) | 15 (21.7) |  |
| Others | 40 (11.0) | 31 (10.7) | 9 (11.8) |  | 40 (10.9) | 30 (10.0) | 10 (14.5) |  |
| **Age at diagnosis (years), n (%)** |  |  |  | 0.239 |  |  |  | **0.017** |
| 2 to < 10 | 260 (71.2) | 210 (72.7) | 50 (65.8) |  | 262 (71.2) | 221 (73.9) | 41 (59.4) |  |
| ≥ 10 | 105 (28.8) | 79 (27.3) | 26 (34.2) |  | 106 (28.8) | 78 (26.1) | 28 (40.6) |  |
| **Sex, n (%)** |  |  |  | **0.005** |  |  |  | 0.082 |
| Male | 208 (57.0) | 154 (53.3) | 54 (71.1) |  | 211 (57.3) | 165 (55.2) | 46 (66.7) |  |
| Female | 157 (43.0) | 135 (46.7) | 22 (28.9) |  | 157 (42.7) | 134 (44.8) | 23 (33.3) |  |
| **WBC, n (%)** |  |  |  | **0.016** |  |  |  | **0.015** |
| < 50 × 10^9^/L | 267 (73.2) | 218 (75.4) | 49 (64.5) |  | 270 (73.4) | 222 (74.3) | 48 (69.6) |  |
| 50–100 × 10^9^/L | 52 (14.2) | 42 (14.5) | 10 (13.1) |  | 53 (14.4) | 47 (15.7) | 6 (8.7) |  |
| ≥ 100 × 10^9^/L | 46 (12.6) | 29 (10.1) | 17 (22.4) |  | 45 (12.2) | 30 (10.0) | 15 (21.7) |  |
| **Immunophenotype, n (%)** |  |  |  | **0.004** |  |  |  | **0.007** |
| B cell | 304 (83.3) | 249 (86.2) | 55 (72.4) |  | 307 (83.4) | 257 (86.0) | 50 (72.5) |  |
| T cell | 61 (16.7) | 40 (13.8) | 21 (27.6) |  | 61 (16.6) | 42 (14.0) | 19 (27.5) |  |
| **Total XV risk, n (%)** |  |  |  | **< 0.001** |  |  |  | **< 0.001** |
| Low | 177 (48.5) | 171 (59.2) | 6 (7.9) |  | 179 (48.6) | 179 (59.9) | 0 (0.0) |  |
| Standard | 159 (43.6) | 112 (38.7) | 47 (61.8) |  | 160 (43.5) | 115 (38.4) | 45 (65.2) |  |
| High | 29 (7.9) | 6 (2.1) | 23 (30.3) |  | 29 (7.9) | 5 (1.7) | 24 (34.8) |  |
| **MRD on day 19** |  |  |  |  |  |  |  | **< 0.001** |
| < 1% | n/a | n/a | n/a |  | 289 (78.5) | 270 (90.3) | 19 (27.5) |  |
| ≥ 1% | n/a | n/a | n/a |  | 75 (20.4) | 25 (8.4) | 50 (72.5) |  |
| No data | n/a | n/a | n/a |  | 4 (1.1) | 4 (1.3) | 0 (0.0) |  |
| **MRD at end of induction, n (%)** |  |  |  | **< 0.001** |  |  |  |  |
| < 0.01% | 295 (80.8) | 270 (93.4) | 25 (32.9) |  | n/a | n/a | n/a |  |
| ≥ 0.01% | 69 (18.9) | 19 (6.6) | 50 (60.8) |  | n/a | n/a | n/a |  |
| No data | 1 (0.3) | 0 (0.0) | 1 (1.3) |  | n/a | n/a | n/a |  |
| **BMI (4 subgroups), n (%)** |  |  |  | 0.428 |  |  |  | 0.177 |
| < 5% | 25 (6.9) | 22 (7.6) | 3 (3.9) |  | 26 (7.0) | 24 (8.0) | 2 (2.9) |  |
| 5% to < 85% | 238 (65.2) | 183 (63.3) | 55 (72.4) |  | 241 (65.5) | 189 (63.2) | 52 (75.4) |  |
| 85% to < 95% | 45 (12.3) | 38 (13.2) | 7 (9.2) |  | 44 (12.0) | 36 (12.1) | 8 (11.6) |  |
| ≥ 95% | 57 (15.6) | 46 (15.9) | 11 (14.5) |  | 57 (15.5) | 50 (16.7) | 7 (10.1) |  |
| **BMI (3 subgroups), n (%)** |  |  |  | 0.278 |  |  |  | 0.113 |
| < 5% | 25 (6.9) | 22 (7.6) | 3 (3.9) |  | 26 (7.1) | 24 (8.0) | 2 (2.9) |  |
| 5% to < 85% | 238 (65.2) | 183 (63.3) | 55 (72.4) |  | 241 (65.5) | 189 (63.2) | 52 (75.4) |  |
| ≥ 85% | 102 (27.9) | 84 (29.1) | 18 (23.7) |  | 101 (27.4) | 86 (28.8) | 15 (21.7) |  |
| **Obese vs. non-obese, n (%)** |  |  |  | 0.758 |  |  |  | 0.173 |
| ≥ 95% | 57 (15.6) | 46 (15.9) | 11 (14.5) |  | 57 (15.5) | 50 (16.7) | 7 (10.1) |  |
| Other | 308 (84.4) | 243 (84.1) | 65 (85.5) |  | 311 (84.5) | 249 (83.3) | 62 (89.9) |  |

Abbreviations: MRD, minimal residual disease; WBC, white blood cells; BMI, body mass index.

**Supplementary Table S3.** Disease outcome according to BMI percentile change^*^

| **Outcomes** | **BMI percentile change*** | | | | |
| --- | --- | --- | --- | --- | --- |
|  | **N** | **Mean** | **Range** | **SE** | ***P*-value** |
| **MRD on day 19** |  |  |  |  | 0.226 |
| < 1% | 289 | 7.41 | −84.60–93.32 | 1.59 |  |
| ≥ 1% | 76 | 3.27 | −59.30–54.21 | 2.76 |  |
| No data | 8 | -0.22 | −61.60–30.02 | 10.69 |  |
| **MRD at end of induction** |  |  |  |  | 0.989 |
| < 0.01% | 299 | 6.72 | −84.60–93.32 | 1.54 |  |
| ≥ 0.01% | 69 | 6.67 | −59.30–66.77 | 3.16 |  |
| No data | 5 | −15.70 | −61.10–7.15 | 11.75 |  |
|  | **Parameter estimate** | | **SE** | | ***P*-value** |
| **CIR** | −0.0001 | | 0.007 | | 0.607 |
| **EFS** | −0.006 | | 0.005 | | 0.190 |
| **OS** | −0.010 | | 0.006 | | 0.117 |

*****BMI percentile change was calculated by subtracting BMI percentile at diagnosis from BMI percentile

at end of induction. Negative values denoted loss of BMI and positive values denoted BMI gain.

Abbreviations: BMI, body mass index; SE, standard error; MRD, minimal residual disease; CIR,

cumulative incidence of refractory disease/relapse; EFS, event-free survival; OS, overall survival.

**Supplementary Table S4.** Patients who experienced events

| **Patient** | **Age at diagnosis (years)** | **Sex** | **BMI category** | **Total XV risk** | **Event** | **Time to relapse* (months)** | **Status** | **Cause of death** |
| --- | --- | --- | --- | --- | --- | --- | --- | --- |
| 1 | 2.0 | M | ≥ 95th | Standard | Isolated CNS relapse | 38 | Alive |  |
| 2 | 10.9 | M | ≥ 95th | Standard | Isolated CNS relapse | 11 | Alive |  |
| 3 | 5.9 | M | ≥ 95th | Low | Isolated CNS relapse | 35 | Alive |  |
| 4 | 4.9 | M | ≥ 95th | High | Hematologic and CNS relapse | 39 | Alive |  |
| 5 | 8.8 | F | ≥ 95th | High | Hematologic relapse | 53 | Expired | Death due to disease |
| 6 | 12.1 | M | ≥ 95th | Standard | Hematologic relapse | 15 | Expired | Death due to disease |
| 7 | 15.9 | F | ≥ 95th | Standard | Hematologic relapse | 4 | Expired | Death due to disease |
| 8 | 14.2 | M | ≥ 95th | Standard | Hematologic and CNS relapse | 14 | Expired | Death due to disease |
| 9 | 16.2 | M | ≥ 95th | Standard | Motor vehicle accident | 38 | Expired | Death due to accident |
| 10 | 4.3 | M | ≥ 95th | Standard | Treatment-related toxicity | N/A | Expired | *B. cereus* sepsis during induction |
| 11 | 6.1 | F | ≥ 95th | Standard | Treatment-related toxicity | N/A | Expired | Transplant-related death |
| 12 | 18.3 | M | ≥ 95th | Low | Treatment-related toxicity | N/A | Expired | Invasive *Clostridium* and *Bacteroides* infection during consolidation |
| 13 | 9.5 | F | ≥ 95th | Standard | Treatment-related toxicity | N/A | Expired | Liver failure during continuation therapy |
| 14 | 6.0 | M | < 5th | Standard | Isolated CNS relapse | 15 | Alive |  |
| 15 | 7.8 | F | 5th to 84th | Standard | Hematologic and extramedullary relapse | 37 | Alive |  |
| 16 | 7.8 | F | 5th to 84th | Low | Hematologic relapse | 54 | Alive |  |
| 17 | 5.3 | M | 5th to 84th | Low | Hematologic relapse | 111 | Alive |  |
| 18 | 2.3 | F | 5th to 84th | Standard | Hematologic relapse | 30 | Alive |  |
| 19 | 2.7 | M | 5th to 84th | Low | Hematologic relapse | 65 | Alive |  |
| 20 | 4.3 | M | 5th to 84th | Standard | Isolated CNS relapse | 22 | Alive |  |
| 21 | 11.2 | M | 5th to 84th | Standard | Isolated CNS relapse | 37 | Alive |  |
| 22 | 5.4 | F | 5th to 84th | High | Isolated CNS relapse | 4 | Alive |  |
| 23 | 7.1 | M | 5th to 84th | Standard | Isolated CNS relapse | 27 | Alive |  |
| 24 | 5.2 | M | 5th to 84th | Standard | Isolated CNS relapse | 22 | Alive |  |
| 25 | 4.9 | M | 5th to 84th | Standard | Hematologic and CNS relapse | 48 | Alive |  |
| 26 | 5.0 | F | 5th to 84th | High | Hematologic and CNS relapse | 12 | Alive |  |
| 27 | 3.2 | F | 5th to 84th | Low | Hematologic and CNS relapse | 76 | Alive |  |
| 28 | 3.3 | M | 5th to 84th | High | Refractory disease | N/A | Alive |  |
| 29 | 18.0 | M | 5th to 84th | High | Refractory disease | N/A | Alive |  |
| 30 | 3.9 | M | 5th to 84th | High | Refractory disease | N/A | Alive |  |
| 31 | 6.4 | M | 5th to 84th | High | Refractory disease | N/A | Alive |  |
| 32 | 7.2 | M | 5th to 84th | Low | Testicular relapse | 49 | Alive |  |
| 33 | 3.4 | M | 5th to 84th | Standard | Testicular relapse | 44 | Alive |  |
| 34 | 5.4 | M | 5th to 84th | Standard | Hematologic relapse | 60 | Expired | Death due to disease |
| 35 | 14.7 | M | 5th to 84th | Standard | Hematologic relapse | 6 | Expired | Death due to disease |
| 36 | 7.8 | M | 5th to 84th | Standard | Hematologic relapse | 9 | Expired | Death due to disease |
| 37 | 7.0 | M | 5th to 84th | Low | Hematologic relapse | 45 | Expired | Death due to disease |
| 38 | 11.9 | F | 5th to 84th | High | Hematologic relapse | 9 | Expired | Death due to disease |
| 39 | 9.5 | M | 5th to 84th | Standard | Hematologic relapse | 60 | Expired | Death due to disease |
| 40 | 3.8 | M | 5th to 84th | Standard | Hematologic relapse | 49 | Expired | Death due to disease |
| 41 | 18.7 | M | 5th to 84th | Standard | Hematologic relapse | 37 | Expired | Death due to disease |
| 42 | 9.8 | M | 5th to 84th | High | Hematologic relapse | 11 | Expired | Death due to disease |
| 43 | 12.2 | M | 5th to 84th | Standard | Hematologic relapse | 9 | Expired | Death due to disease |
| 44 | 3.9 | M | 5th to 84th | High | Hematologic relapse | 22 | Expired | Death due to disease |
| 45 | 10.0 | M | 5th to 84th | Standard | Hematologic relapse | 14 | Expired | Death due to disease |
| 46 | 10.4 | M | 5th to 84th | High | Refractory disease | N/A | Expired | Death due to disease |
| 47 | 11.6 | M | 5th to 84th | High | Treatment-related toxicity | N/A | Expired | Transplant-related death |
| 48 | 2.7 | M | 5th to 84th | Low | Treatment-related toxicity | N/A | Expired | *B. cereus* sepsis during induction |
| 49 | 11.6 | F | 5th to 84th | High | Treatment-related toxicity | N/A | Expired | Transplant related death |
| 50 | 10.1 | F | 85th to 94th | Standard | Hematologic relapse | 57 | Alive |  |
| 51 | 11.5 | F | 85th to 94th | Low | Hematologic relapse | 8 | Expired | Death due to disease |
| 52 | 3.5 | M | 85th to 94th | Standard | Hematologic relapse | 23 | Expired | Death due to disease |
| 53 | 12.6 | F | 85th to 94th | Standard | Second malignancy | N/A | Expired | Death due to second malignancy (high-grade glioma) |
| 54 | 13.3 | F | 85th to 94th | Standard | Second malignancy | N/A | Expired | Death due to second malignancy (acute myeloid leukemia) |
| 55 | 14.4 | M | 85th to 94th | High | Treatment-related toxicity | N/A | Expired | Transplant-related death |

* Time from diagnosis to relapse in months

Abbreviations: BMI, body mass index; CNS, central nervous system; N/A, not applicable
